# Glioblastoma 20 years after a nail gun trauma: A risk factor?

**DOI:** 10.1002/ccr3.5927

**Published:** 2022-06-02

**Authors:** Megan M. Finneran, Michael Young, Larry Joyce, Emilio M. Nardone

**Affiliations:** ^1^ 71747 Neurological Surgery Carle BroMenn Medical Center Normal Illinois USA; ^2^ Department of Pathology OSF St. Joseph Medical Center Bloomington Illinois USA

**Keywords:** foreign body, glioblastoma, nail gun, penetrating head injury, post‐traumatic

## Abstract

A 48‐year‐old man presented with headaches and confusion. Imaging demonstrated a right frontal glioblastoma multiforme (GBM), twenty years after a nail gun injury to the same region. GBM in the same location as a previous injury points toward possible causation from the trauma in the development of a high‐grade glioma.

## Graphical abstract

The link between trauma and development of high‐grade glioma is suspected but not well‐established. We present a case of a 48‐year‐old man with GBM in the location as a nail gun injury 20 years prior.

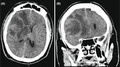

## 1 INTRODUCTION

Several studies have looked at the relationship between traumatic brain injury and the development of brain tumors.^1^, ^2^, ^3^, ^4^, ^5^, ^6^, ^7^ However, there has been little research about penetrating brain injury as a causation in the development of brain tumors. There have been few case reports of local penetrating injury and the relationship of post‐traumatic brain tumors, specifically gliomas.^8^, ^9^, ^10^, ^11^, ^12^, ^13^, ^14^, ^15^, ^16^, ^17^, ^18^, ^19^ These reports all have the same anatomic location as the site of the injury and glioblastoma multiforme (GBM), further raising the hypothesis of causation in penetrating brain injury and GBM. We present a case of self‐inflicted accidental nail gun injury in the right orbitofrontal region with development of GBM twenty years later in the same location of the nail gun injury.

## 2 CASE REPORT

### 2.1 History

A 48‐year‐old man with past medical history of hypertension and prior head injury presented to the emergency department after he drove his tractor off the side of the road. His brother stated that the patient had exhibited confusion over the previous several months. The patient reported occipital headaches, dizziness, and several falls over the preceding 3–4 weeks.

**FIGURE 1:**
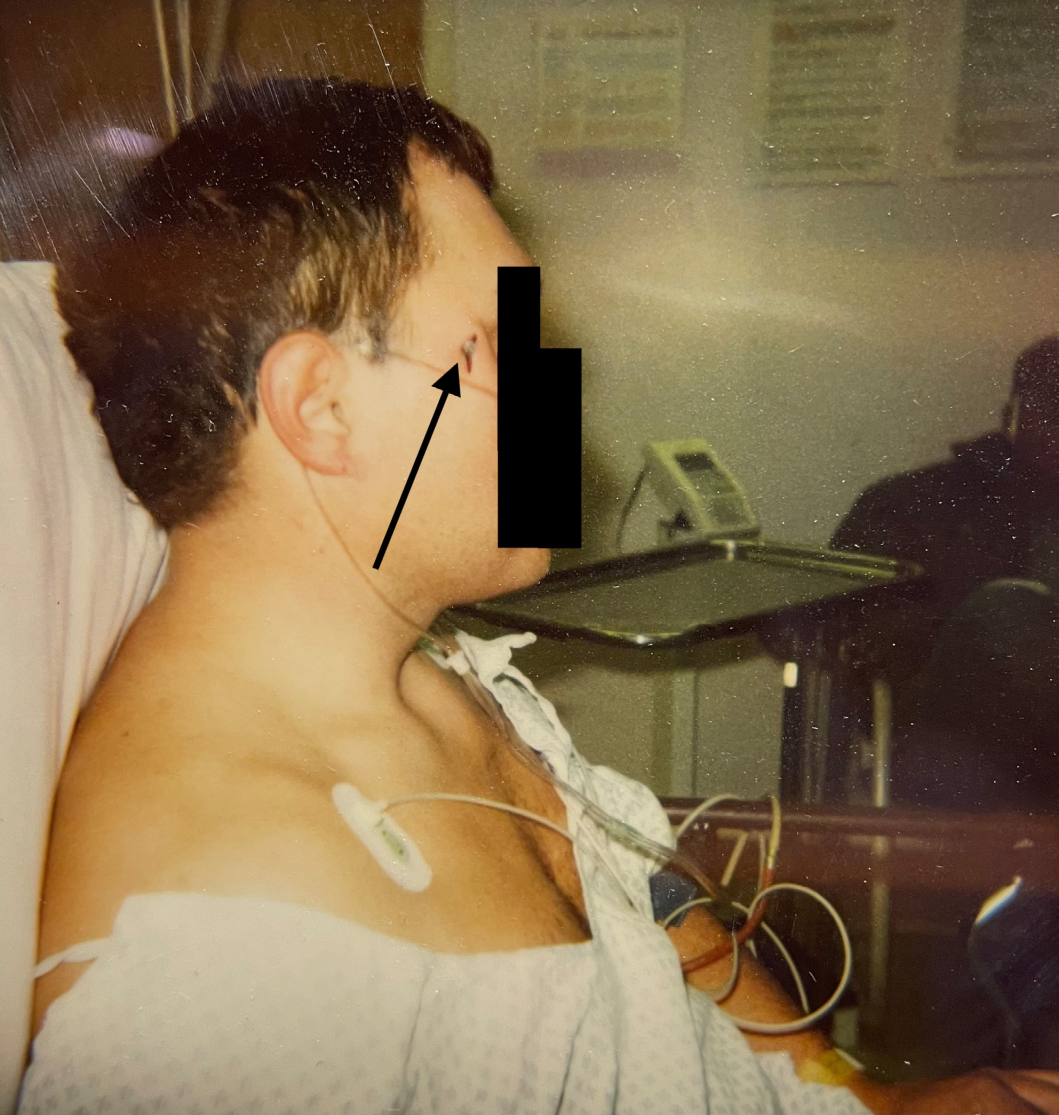
A lateral photograph displays the patient in 2001 after a self‐inflicted accidental nail gun injury. The nail is visualized lateral to the right lateral canthus (black arrow)

**FIGURE 2:**
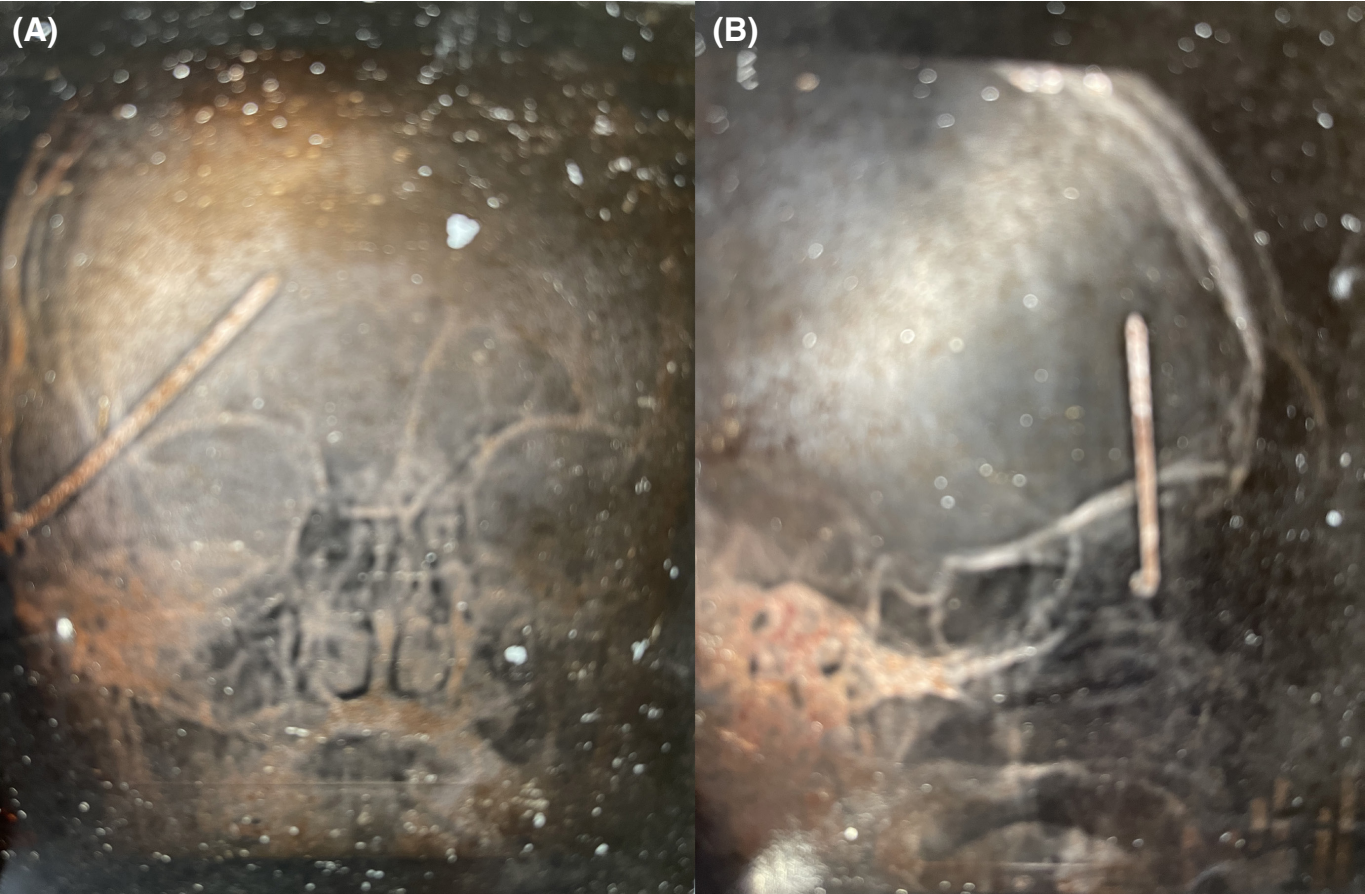
Anteroposterior (A) and lateral (B) radiographs reveal the superomedial trajectory of the nail into the brain parenchyma. The patient had been carrying the radiograph prints in his wallet for twenty years

Upon further discussion, the patient described a work‐related unintentional self‐inflicted nail gun injury with entry just lateral to the right lateral canthus twenty years prior (Figure 1). At that time, the patient presented to the emergency department awake and alert with no neurological deficits. Skull radiographs showed the superomedial trajectory of the nail through the skull into the right frontal brain parenchyma (Figure 2). He was taken to the operating room and the patient was put under general anesthesia and prepared for a possible craniotomy. Ultimately, the nail was easily extracted in total with a clamp applied to the screw head that was the only portion of the nail visible at the level of the skin. There were no complications and the patient recovered well. There was concern for a small amount of residual metal near the right orbit and the patient had been told he could not undergo magnetic resonance imaging (MRI).

### 2.2 Examination

On examination, he had no neurological deficits; cranial nerves were intact, speech was fluent, and he had full strength in all muscle groups.

### 2.3 Imaging

**FIGURE 3:**
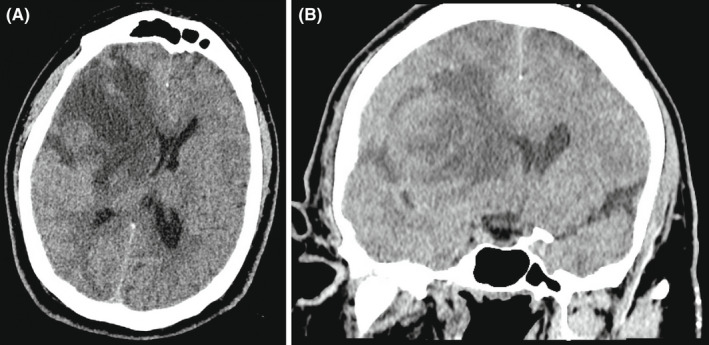
Axial (A) and coronal (B) head computed tomography (CT) without contrast demonstrates a right frontal lesion causing mass effect and midline shift

**FIGURE 4:**
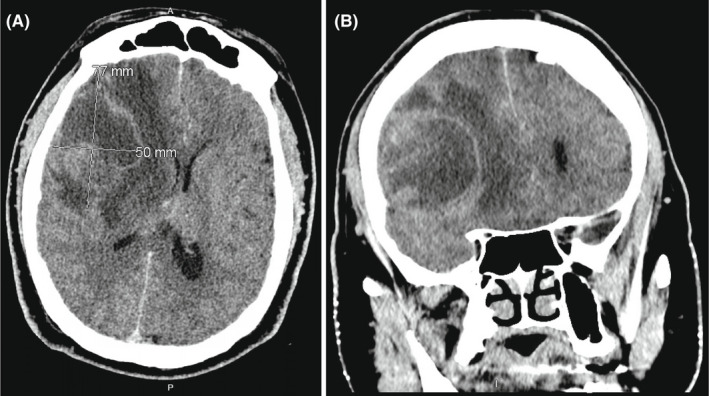
Axial (A) and coronal (B) head CT with contrast outlines a partially and peripherally enhancing right frontal lesion, measuring 77 × 50 × 61mm

Head CT without contrast demonstrated a large right frontal intra‐axial lesion with vasogenic edema and 12mm of midline shift (Figure 3) in the same location as the initial nail gun injury. A head CT with contrast showed peripheral enhancement of the 77mm x 50mm x 61mm mass (Figure 4).

### 2.4 Hospital course

**FIGURE 5:**
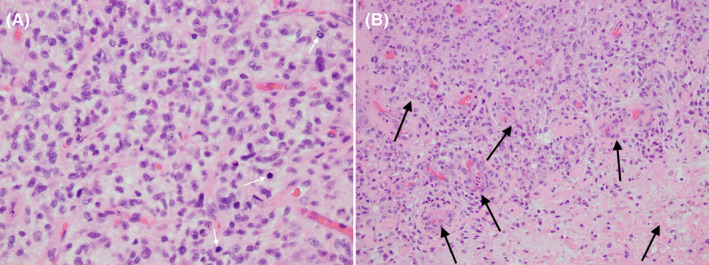
Hematoxylin and eosin stain show glioblastoma with mitotic figures (A, 400X, white arrows) and microvascular proliferation (B, 200X, black arrows)

He was started on dexamethasone 6mg every six hours and levetiracetam 500mg every twelve hours. A CT of the chest, abdomen, and pelvis was negative for lesion. Three days after presentation, the patient underwent a right frontal craniotomy with neuronavigation and gross total resection of the tumor was achieved. Pathology revealed high‐grade primary glial neoplasm consistent with glioblastoma (Figure 5), IDH‐wild type with positive O^6^‐methylguanine‐DNA‐methyltransferase (MGMT) promoter methylation. He was discharged home on post‐operative day two.

### 2.5 Treatment

Six weeks post‐operatively, treatment consisted of temozolamide 75 mg/m^2^ daily with concurrent partial brain intensity‐modulated radiotherapy at a dose of 60 Gy in 30 fractions. This was followed by temozolamide 200 mg/m^2^ days one through five, cycled every 28 days for six cycles. Tumor‐treating fields (TTF) therapy was offered but the patient declined. Six months post‐operatively, the patient was doing well and tolerating treatment.

## 3 DISCUSSION

Guidelines for classification of post‐traumatic glioma were originally proposed by Zulch et al in 1965 and include (1) the patient was in good health prior to the injury; (2) the injury must be serious enough to cause brain contusion and a secondary repair process; (3) the location of the injury and the tumor should directly correspond; (4) there should be at least one year time interval between the injury and appearance of the tumor; (5) there must be histological proof of tumor pathology; (6) trauma should involve an external force.^20^

In 1972, Manuelidis added the following criteria: (1) the traumatized brain must be demonstrated histologically; (2) bleeding, scar, and edema due to the presence of tumor must be differentiated from similar features caused by trauma; (3) tumor tissue should be directly continuous with scar from the trauma, not simply close to it.^21^

These criteria were defined in the pre‐CT era, and thus, changes have been proposed to eliminate or supplement the need for histological confirmation with imaging findings.^17^ However, no official change in the criteria has been implemented to reflect the evolution of imaging.

Inflammation and oxidative stress have been reported in both traumatic brain injury (TBI) and glioma.^19^ Throughout the body acutely after a trauma, mobilization of microglia, myeloid inflammatory cells, peripheral neutrophils, monocytes, and eosinophils occurs as they are recruited to the site of injury.^22^ Inflammatory cells can then contribute to oncogenesis through reactive oxygen species.^23^ In the brain in particular, microglia have an active role in the inflammatory process.^24^ Inflammatory cytokines also play a role and have shown to be upregulated in TBI, which are increasingly induced by interleukin‐6 (IL‐6).^19^ The increase in IL‐6 production by glial cells increases vascular permeability and damages the blood‐brain barrier.^24^ It is predicted that GBM may results as neural stem cells or progenitor cells migrate to the site of injury in a quest to repair damaged tissue.^23^

### 3.1 Observations

We report the first case of post‐traumatic GBM after a penetrating nail gun injury. Thirteen cases of post‐traumatic high‐grade glioma have been previously reported (Table 1). In each case, the tumor occurred in the same region as the original injury. The time frame between trauma and identification of lesion ranged from four years to 38 years. Eleven of the cases reported GBM, one reported a mixed glioma, and one an astrocytoma grade III.

**TABLE 1:** Thirteen previous case reports of post‐traumatic glioma are described

Case	Author/Year	Sex	Age (at time of Lesion)	Injury Mechanism	Time Between Injury and Lesion	Lesion Pathology	Location
1	Mrowka et al/1978	unknown	unknown	Artillery projectile	30 years	GBM	L parietal
2	Witzmann et al/1981	M	33	GSW	5 years	GBM	Bifrontal
3	Schmitt/1983	M	75	Shell splinters WW2	38 years	GBM	Bifrontal
4	Troost, Tulleken/1984	M	unknown	Bombshell WW2	unknown	Astrocytoma grade III	unknown
5	Di Trapani et al/1996	unknown	unknown	Commutive trauma	Several years	Mixed glioma	L parietal
6	Sabel et al/1999	M	55	Penetrating metal splinter (homemade firework)	37 years	GBM	L frontal
7	Magnavita et al/2003	M	unknown	Car accident, no radiographic findings	4 years	GBM	R temporo‐parietal
8	Moorthy, Rajshekhar/2004	M	56	Head injury, b/l basifrontal contusions	5 years	GBM	L frontal
9	Anselmi et al/2006	M	40	Road accident w/ICH	20 years	GBM	R parietal
10	Zhou, Liu/2010	M	45	Road accident w/ICH	10 years	GBM	R temporal
11	Han et al/2013	F	29	Motorbike accident	9 years	GBM	R frontal
12	Tyagi et al/2016	M	65	Fall, L frontal contusion	11 years	GBM	L frontal
13	Tyagi et al/2016	M	54	MVC w/contusion	7 years	GBM	Inferior R frontal

Injury etiology included an artillery projectile,^8^ a gunshot wound,^9^ shell splinters from World War II,^10^ a bombshell from World War II,^11^ a penetrating metal splinter from a homemade firework,^13^ a car accident with no radiographic findings,^14^ two road accidents resulting in intracerebral hemorrhage,^16^, ^17^ a motorbike accident,^18^ a fall with identified contusion,^19^ a motor vehicle accident with identified contusion,^19^ and two unspecified traumatic head injuries.^12^, ^15^

Previous epidemiological studies have failed to find conclusive evidence for or against a causative relationship between trauma and tumor occurrence.

Hochberg et al^1^ performed a case‐control study of 160 patients diagnosed with GBM and 128 controls. Data were gathered by questionnaire and participants were asked if they had experienced a “severe” head injury at age 15 or later. They found a significantly increased risk for development of GBM with history of severe head trauma.

Zampieri et al^2^ compared 195 cases of glioma to matched controls with a questionnaire, including inquiries about education, medical history, radiation exposure, head trauma, blood type, and family history. The study found no conclusive associations between trauma and tumor occurrence.

A case–control study by Preston‐Martin et al^3^ compared 1178 glioma and 330 meningioma cases to matched controls and asked about history of head injury at least five years before diagnosis that required medical attention. They found no association for glioma but found increased odds ratios for men with meningiomas.

Hu et al^4^ conducted a case–control study among 218 cases of glioma and 436 controls. Their questionnaire results highlighted increased odds ratio for head trauma and decreased glioma risks in those patients with increased consumption of fruits and vegetables.

Inskip et al^5^ conducted a population‐based study among more than 200,000 Danish residents hospitalized due to concussion, skull fracture, or other head injury. It showed an increased overall incidence of intracranial tumors in head trauma patients, but no significant association was found for malignant astrocytic tumors. However, the majority of intracranial tumors were discovered during the first year post‐injury, raising the possibility of the presence prior to the injury. Additionally, average follow‐up was eight years, with a maximum of 17 years. It is entirely possible there was occurrence among patients beyond the authors’ follow‐up period.

Nygren et al^6^ performed a similar population‐based study of more than 300,000 patients hospitalized for TBI in Sweden. A total of 281 brain tumors were diagnosed through register assessments during the follow‐up period. The authors found no association between TBI and the risk of primary brain tumor. No stratification was performed based on severity of injury.

A population study based in Taiwan by Chen et al^7^ yielded different results. The study cohort of more than 5000 patients diagnosed with TBI was compared to a random cohort of 25,000 people. Over a three‐year follow‐up period, nine patients from each cohort developed a primary brain tumor, and those in the TBI cohort were more likely to have a diagnosis of malignant brain tumor. The authors found an association between TBI severity and malignant brain tumor occurrence.

There have been no new studies evaluating the relationship between trauma and brain tumor for the past twenty years, highlighting the need to reinvestigate a possible link. Additionally, none of the mentioned studies define the proximity of tumor to area of traumatic injury. Therefore, it is difficult to ascertain which tumors were truly post‐traumatic in nature. Of the existing case reports in the literature, including the nail gun injury reported here, it seems possible that a penetrating head injury with a foreign body and its associated scar tissue would increase the risk of post‐traumatic glioma. This remains to be definitively evaluated.

The authors suggest modifying the criteria to eliminate the histologic demonstration of traumatized brain criteria proposed by Manuelidis; this can be replaced with “the traumatized brain must be demonstrated radiographically.” Given the relative paucity of reported cases and available literature, it may also be prudent to include an ICD‐10 code for diagnosis of post‐traumatic glioma. This would allow the neurosurgical and medical community as a whole to better capture these cases in order to further explore the relationship between trauma and subsequent pathology.

## 4 CONCLUSIONS

We present a rare case of a patient who developed an accidental, self‐inflicted nail gun injury to the right orbitofrontal skull and underlying brain parenchyma with development of GBM in the site of the nail gun injury 20 years later. This case report and the few others in the literature suggest a causative relationship between penetrating traumatic injury and high‐grade glioma. Further studies are needed to continue to investigate head trauma as a possible risk factor for GBM.

## AUTHOR CONTRIBUTIONS

Megan Finneran: Composed manuscript, gathered figures. Michael Young: Edited manuscript, contributed to manuscript. Larry Joyce: Collected pathology slides and contributed captions. Emilio Nardone: Primary surgeon, edited manuscript.

## CONFLICT OF INTEREST

The authors have no conflict of interest to disclose.

## CONSENT

Written informed consent was obtained from the patient to publish this report in accordance with the journal's patient consent policy.

## Data Availability

The data that support the findings of this study are available on request from the corresponding author. The data are not publicly available due to privacy or ethical restrictions.
